# Novelty in Impact of Neurorehabilitation in a Classic Case of Syringomyelia

**DOI:** 10.7759/cureus.29126

**Published:** 2022-09-13

**Authors:** Sanika P Gade, Pallavi Harjpal, Rakesh K Kovela

**Affiliations:** 1 Physiotherapy, Ravi Nair Physiotherapy College, Datta Meghe Institute of Medical Sciences, Wardha, IND; 2 Physiotherapy, NITTE Institute of Physiotherapy, NITTE (Deemed to be University), Mangalore, IND

**Keywords:** strength training, case report, physiotherapy rehabilitation, syringomyelia, arnold chiari malformation

## Abstract

A fluid-filled hole inside the parenchyma or central canal of the spinal cord causes syringomyelia, a neurological condition. It is most frequently linked to type 1 Chiari malformations. Syringomyelia can be caused by tumors in the spinal cord, trauma, and post-traumatic or infectious adhesive arachnoiditis. Syringomyelia is shown to have a prevalence of 8.4/100,000 to 0.9/10,000 in certain studies, making it one of the few unusual cases. A large proportion of patients are between 20 and 50 years of age. In our case, the patient is a 17-year-old boy who complained of tingling and weakness in both lower extremities, as well as loss of sensation in both hands. MRI of his spine revealed a Chiari I malformation involving evidence of medulla, fourth ventricle, and cerebellar vermis displacement into the foramen magnum. Arnold Chiari's malformation with cord syringomyelia and tonsillar herniation was diagnosed based on the symptoms and investigation findings. The goal of this case is to highlight the benefits of exercise treatment in improving the patient's quality of life, as physiotherapy protocol instillation is not practiced on a daily basis for such conditions.

## Introduction

Syringomyelia is a condition in which a fluid-filled cyst (syrinx) forms inside your spinal cord. Over time, the cyst might become larger, causing damage to your spinal cord and symptoms such as pain, numbness, and stiffness. Syringomyelia has several possible causes, but the majority of cases are associated with Chiari malformation, a condition in which the brain matter protrudes into the spinal canal. The Chiari malformation is a collection of posterior fossa malformations characterized by cerebellar tonsillar descent through the foramen magnum [1]. It was first discovered about a century earlier. Although the surgical approach varies, the goals of surgery are to relieve brainstem compression and cranial nerve distortion, restore normal CSF flow across the foramen magnum, and reduce the size of any associated syrinx cavity [2]. Although Chiari anomalies are among the most frequent cause of syrinx cavities, spinal syringomyelia can also be caused by traumatic, viral, degenerative, and other pathophysiology that causes a partial CSF flow restriction in the spinal subarachnoid space [3].

The growth of syringomyelia linked with Chiari I malformation is caused by the action of the cerebellar tonsils, which partially occlude the subarachnoid space at the foramen magnum and act as a piston on the partially contained spinal subarachnoid space [4]. Elevated cervical subarachnoid pressure waves compress the spinal cord from the outside rather than the inside, causing syrinx fluid to travel caudally with each pulse, resulting in syrinx progression. The abnormal structure and location of the tonsils disappear after simple decompressive extra-arachnoid surgery, demonstrating that the Chiari I malformation of the cerebellar tonsils is acquired rather than congenital [5]. Myelopathy worsens as a result of syringomyelia. Most syringomyelia patients' cerebellar tonsils have a Chiari I defect [6]. Syringomyelia, which affects your back, shoulders, arms, and legs, causes muscle weakening and wasting (atrophy), headaches, loss of sensitivity to pain and warmth, stiffness in the back, shoulders, arms, and legs, and discomfort in the neck, arms, and back. In children with syringomyelia, scoliosis or other postural deformities are common and substantial [7]. Physiotherapy is broadly utilized and has an important role in the treatment of physical, mental, and social problems [8]. Physiotherapy is underutilized due to a scarcity of data, expertise, and resources; thus, this article emphasizes the relevance of physical therapy in symptom improving and maintaining patient quality of life.

## Case presentation

A 17-year-old child, a resident of Yavatmal, presented with complaints of tingling sensation and weakness in bilateral lower limbs along with paresthesia in bilateral hands for five months. The patient gave a history of fall from a bullock cart six months ago. The symptoms were insidious in onset and gradually progressive. There was no history of loss of consciousness, ENT bleeding, vomiting or nausea, or seizures. He came to our hospital with the above-mentioned history and was suggested to undergo CT of the cervical spine. There was evidence of long segment central spinal cord hypodensity involving cervical cord of length >12 cm, for which he underwent foramen magnum decompression with tonsillar elevation; C1 laminectomy. Post-operatively, the patient was shifted to neuro-ICU where he was managed with antibiotics, analgesics, antiepileptic, and other supportive measures. He was then shifted to the neurosurgery ward after being neurologically and hemodynamically stable.

Clinical findings

**Figure 1:**
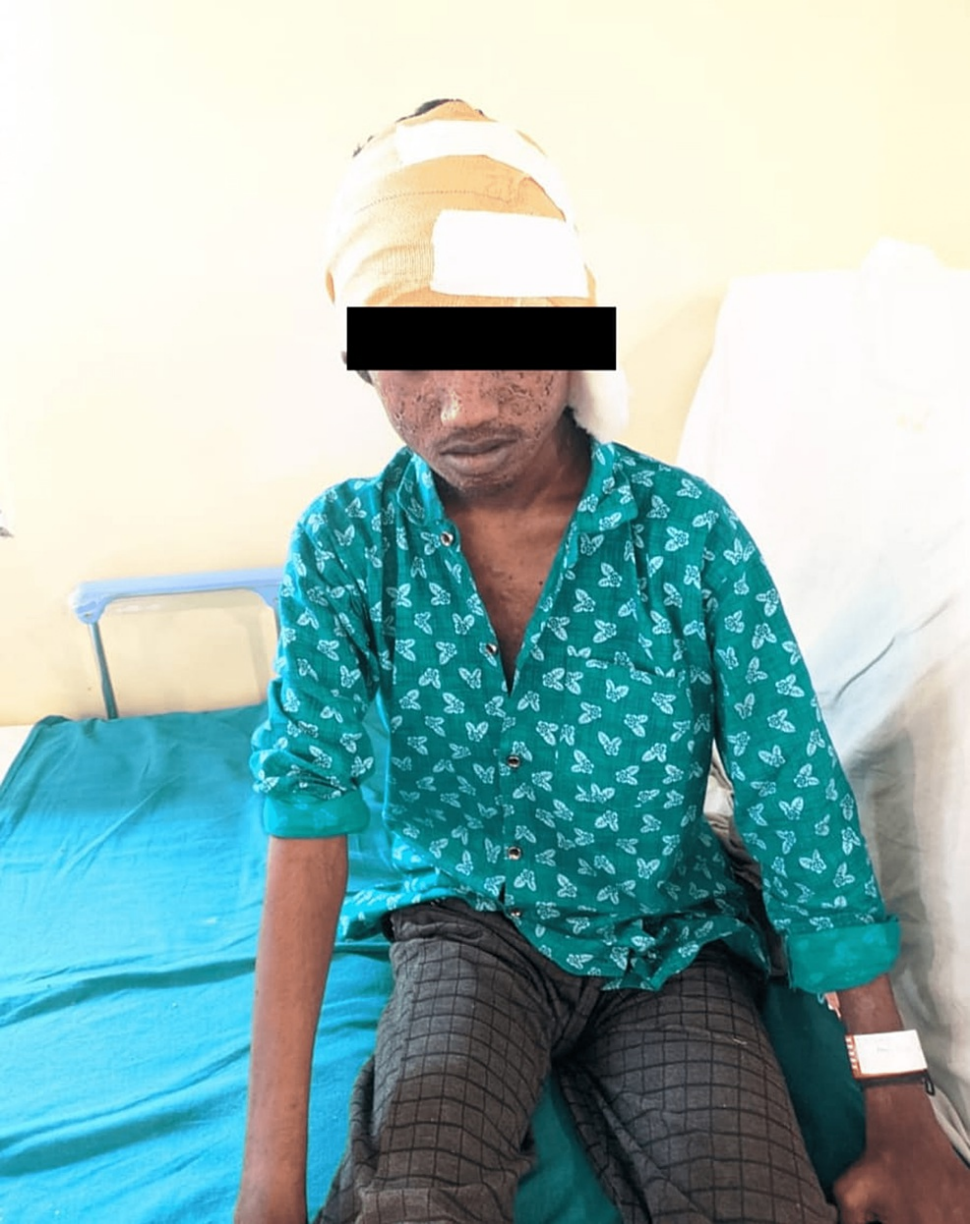
Dermatological changes on the face.

On post-operative day 3, the patient was visited by the physiotherapist in the neurosurgery ward. He was seen in an upright position and was conscious, cooperative, and oriented to time, place, and person. On observation, hip and knee were in a neutral position and ankle in plantarflexion; in the standing position, posture was assessed, which revealed increased kyphosis and functional scoliosis. Dermatological changes over the face were evident, as shown in Figure 1. On examination, muscle weakness on the bilateral lower limb was present and was graded as 3/5 on manual muscle testing, and neural tension test for sciatic nerve was positive for both the lower extremities, which were probable causes for the tingling sensation. Superficial sensations including temperature and pain were diminished in bilateral hands at C6, C7, C8, and T1 levels.

Diagnostic assessment

**Figure 2:**
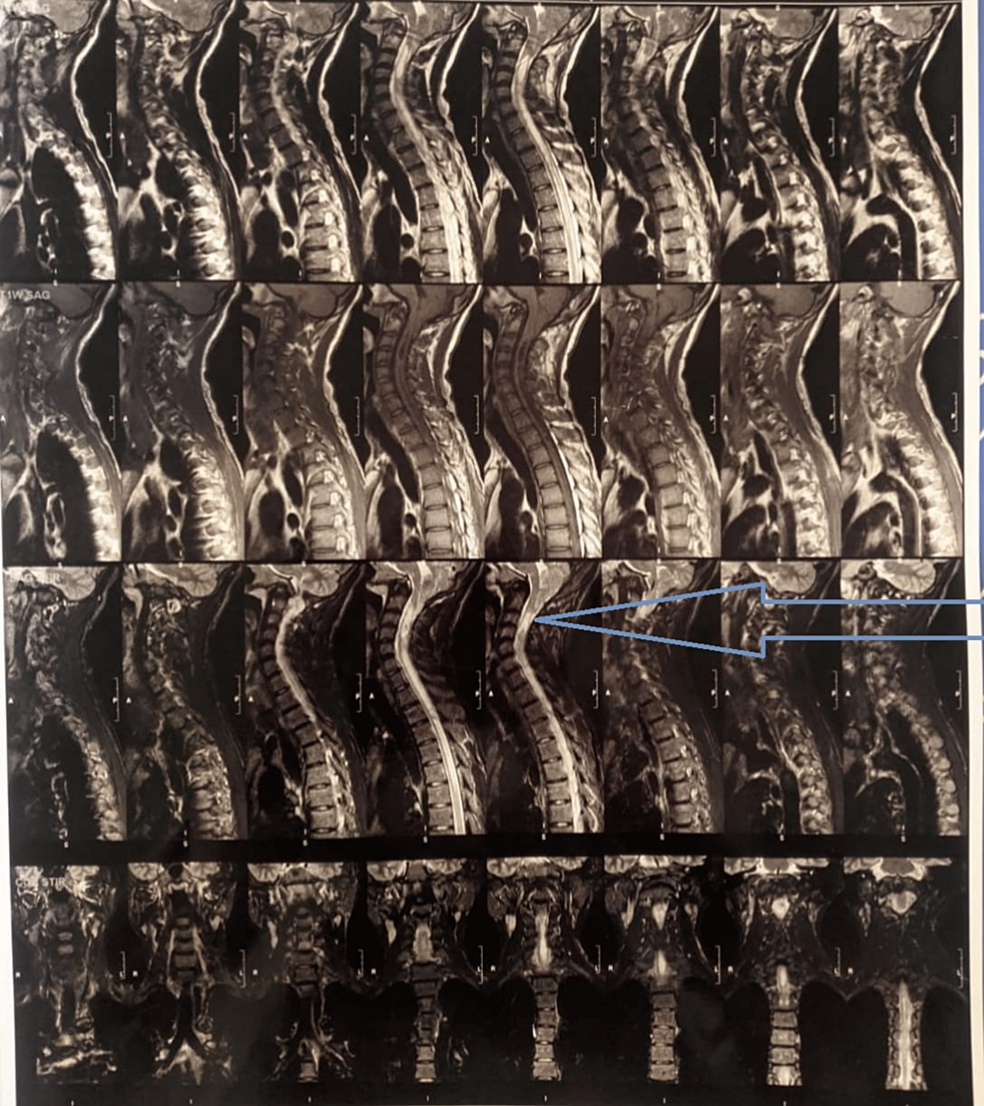
MRI of the spine showing evidence of displacement of the medulla, fourth ventricle, and cerebellar vermis through the foramen magnum.

MRI of the spine was suggestive of Chiari I malformation, showing evidence of displacement of the medulla, fourth ventricle, and cerebellar vermis through the foramen magnum, as shown in Figure 2.

Treatment interventions

The treatment protocol was planned for the patient in order to relieve his symptoms and improve his quality of life. The protocol followed is mentioned in Table 1.

**Table 1:** Treatment protocol planned for the patient.

	Treatment Goals	Therapeutic Interventions	Dosage
Week 1	To educate the patient and his family members about his condition. To improve strength of affected muscles. To release the tightness of affected muscles.	At the beginning of physical therapy program, the patient and his parents were educated about the condition and its progression, as well as the benefits of exercises. Strengthening was done by giving progressive resistant exercises via half kg weight cuff to bilateral muscles of lower extremities. Stretching exercises to gastrocnemius, soleus, and hamstrings muscles.	10 repetitions x 1 set, 3 repetitions with 30 seconds hold.
Week 2	To improve strength of affected muscles. To release the tightness of affected muscles. To alleviate paresthesia. To regain sensations of pain and temperature.	Strengthening was done by giving progressive resistant exercises via 1-kg weight cuff to bilateral muscles of lower extremities. Stretching exercises to gastrocnemius, soleus, and hamstrings muscles. Neural mobilization for the sciatic nerve to release the compressed nerve and relieve paresthesia. The method used is joint mobilization, mobilization, and release of the sciatic nerve itself. Nerve release helps resolve adhesions and restore normal mechanical function by flossing or mobilizing the affected nerves causing symptoms. Sensory retraining activities were implemented to aid in the restoration of the brain's ability to interpret sensory input. The patient was asked to touch various objects, which stimulates the brain and helps it to rewire itself. Table contact therapy was one of the sensory stimulation activities. Soft scarves, rough sandpaper, fluffy cotton balls, and harsh Velcro were among the items to be grabbed. Without being able to see the objects, the patient was required to pick them up and feel them to discriminate between textures. Soaking a cloth in cold water and another cloth in warm (but not hot) water was another exercise that help people regain their sense of temperature. The therapist then wrapped a cold cloth across his arm. Take in the sensation. Replace the cold towel with the heated cloth after 30 seconds. Try to detect the temperature difference. The patient is then instructed to close his eyes and place one rag on his arm to see if he feels hot or cool.	10 repetitions x 2 sets, 5 repetitions with 30 seconds hold bilaterally. 10 repetitions x 2 sets bilaterally, 15 repetitions x 6 sets per day
Week 3	To improve strength of affected muscles. To release the tightness of affected muscles. To alleviate paresthesia. To regain sensations of pain and temperature. To correct postural abnormalities.	Strengthening was done by giving progressive resistant exercises via 1-kg weight cuff to bilateral muscles of lower extremities. Stretching exercises to gastrocnemius, soleus, and hamstrings muscles. Neural mobilization for the sciatic nerve to release the compressed nerve and relieve paresthesia. Sensory retraining exercises were inculcated, which help restore the brain’s ability to interpret senses. Posture correction exercises included shoulder retraction exercises to reduce increased kyphosis and Schroth method exercises to treat scoliosis.	10 repetitions x 3 sets, 5 repetitions with 30 seconds hold bilaterally. 10 repetitions x 2 sets. 20 repetitions x 6 sets. 20 repetitions x 2 sets each.

The patient and family members were assured that the patient's condition was steadily recovering. The patient and his family/caregivers were taught a home exercise program. It included stretching of gastrocnemius and soleus muscles, hamstrings, and dynamic quadriceps, and continuing with strengthening exercises at home under the supervision of a family member, along with sensory retraining exercises and posture correcting exercises.

Outcome measures

**Figure 3:**
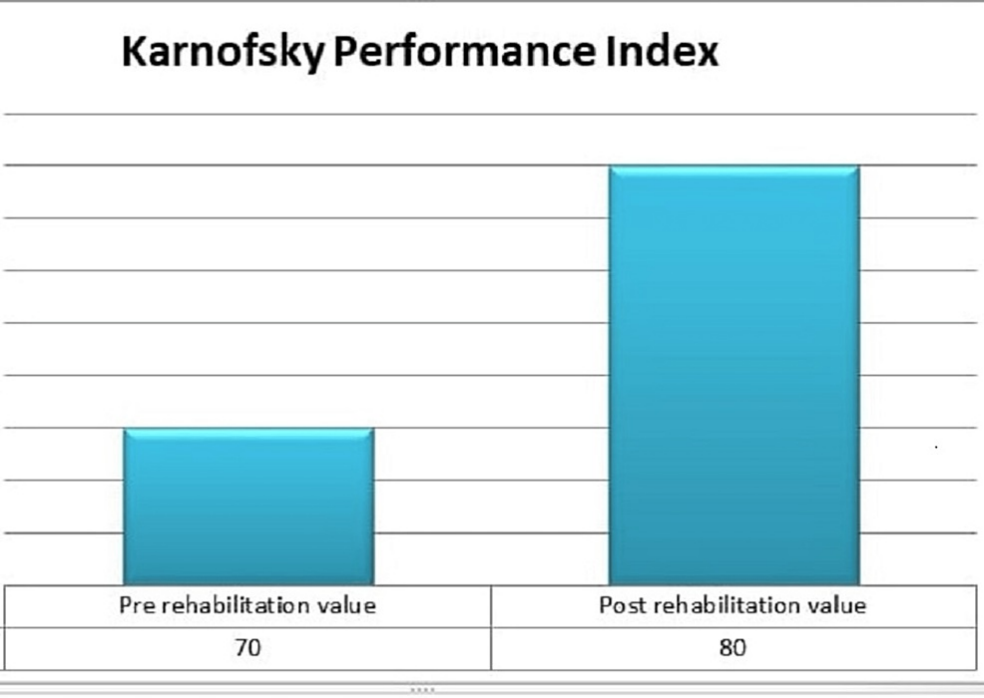
Pre-rehabilitation, the value on the basis of the Karnofsky performance index was 70/100, which indicates that the patient was able to perform self-care activities but used to face difficulty while performing normal daily routine activities; after completion of protocol, the score progressed to 80/100, which indicated that the patient is able to do daily activities apparently and symptoms were prominent only on exertion.

After following the protocol for consecutive 20 days, there was noticeable improvement in the patient’s condition. Outcome measure utilized for this case was the Karnofsky performance index, which is a functional disability evaluation measure that is used to compare the efficacy of various medications and to assess the prognosis of patients. Comparison between pre-rehabilitation and post-rehabilitation is given in Figure 3.

## Discussion

Syringomyelia is a neurologic illness caused by the existence of a fluid-filled cavity within the spinal cord parenchyma or central canal. When type 1 Chiari anomalies are detected, it is most likely. Arnold-Chiari or Chiari anomalies affect the cerebellum, pons, and medulla oblongata. These malformations can produce problems pertaining from cerebellar tonsillar herniation through the foramen magnum to cerebellar absence, and they can occur in combination with other intracranial or extracranial disorders such as hydrocephalus, encephalocele, syrinx, or spinal dysraphism [9]. Syringomyelia is caused by tumors in the spinal cord, as well as trauma and post-traumatic or infectious adhesive arachnoiditis. Although sensory indications such as pain and temperature sensitivity can occur with syringomyelia, it is more usually discovered by chance [10]. According to certain research, the incidence of syringomyelia ranges from 8.4 per 100,000 to 0.9 per 10,000, depending on ethnicity and geography. A large percentage of people are between 20 and 50 years of age [11]. The pressure of the syrinx on the spinal cord commonly causes gradual weakness and stiffness in the back, shoulders, and arms, impairment of pain sensation, difficulty walking, bowel and bladder control problems, pain and numbness of the face, and scoliosis deformity [12]. Syringomyelia is characterized by a steadily increasing cavity in the central spinal canal. This CSF-filled "syrinx" compresses the spinothalamic tract neurons decussating in the anterior white commissure. The posterior columns, on the other hand, are spared due to their distal location. Pain and temperature sensations are lost, but touch and vibratory senses are intact (segmental dissociated sensory loss). In a "cape-like" distribution, the upper limbs are disproportionately involved. Many surgical options for treating syringomyelia exist, including posterior fossa decompression, shunting from the syrinx to the cerebellopontine angle cistern, excision of extramedullary blockages at the rostral end of non-communicating syrinxes, and syringocisternal shunting [13]. Physical therapy has been found to help pain treatment and improve general quality of life in people with syringomyelia. Patient and parent education about the condition and advantages of physical exercises is also part of the rehabilitation program. Stretching and hydrotherapy have been demonstrated to be useful for pain and stiffness reduction. Furthermore, physiotherapy is said to have similar benefits to surgical intervention [14].

## Conclusions

We conclude that a comprehensive strategy comprising conventional treatment and a well-designed physical therapy plan resulted in a considerable improvement in the patient's condition by decreasing symptoms and increasing the patient's quality of life who was diagnosed with Arnold Chiari malformation with cord syringomyelia with tonsillar herniation. The efficacy of a thoroughly monitored physiotherapy program in enhancing the strength and reducing symptoms of the patient who underwent surgery is described in this case analysis.
